# Active Transportation on a Complete Street: Perceived and Audited Walkability Correlates

**DOI:** 10.3390/ijerph14091014

**Published:** 2017-09-05

**Authors:** Wyatt A. Jensen, Barbara B. Brown, Ken R. Smith, Simon C. Brewer, Jonathan W. Amburgey, Brett McIff

**Affiliations:** 1 Department of Family & Consumer Studies, University of Utah, 225 S 1400 E RM 228, Salt Lake City, UT 84112, USA; wyattjensen@gmail.com; 2 Department of Family & Consumer Studies and Cancer Control & Population Sciences, Huntsman Cancer Institute, University of Utah, 225 S 1400 E RM 228, Salt Lake City, UT 84112, USA; ken.smith@fcs.utah.edu; 3 Department of Geography, University of Utah, 332 S 1400 E RM 217, Salt Lake City, UT 84112, USA; simon.brewer@geog.utah.edu; 4 Department of Psychology, Westminster College, 1840 S 1300 E, Salt Lake City, UT 84105, USA; jamburgey@westminstercollege.edu; 5 Utah Department of Health, 288 N 1460 W, Salt Lake City, UT 84116, USA; bmciff@gmail.com

**Keywords:** audited walkability, perceived walkability, complete street, physical activity, active travel, accelerometer, global positioning system

## Abstract

Few studies of walkability include both perceived and audited walkability measures. We examined perceived walkability (Neighborhood Environment Walkability Scale—Abbreviated, NEWS-A) and audited walkability (Irvine–Minnesota Inventory, IMI) measures for residents living within 2 km of a “complete street”—one renovated with light rail, bike lanes, and sidewalks. For perceived walkability, we found some differences but substantial similarity between our final scales and those in a prior published confirmatory factor analysis. Perceived walkability, in interaction with distance, was related to complete street active transportation. Residents were likely to have active transportation on the street when they lived nearby and perceived good aesthetics, crime safety, and traffic safety. Audited walkability, analyzed with decision trees, showed three general clusters of walkability areas, with 12 specific subtypes. A subset of walkability items (*n* = 11), including sidewalks, zebra-striped crosswalks, decorative sidewalks, pedestrian signals, and blank walls combined to cluster street segments. The 12 subtypes yielded 81% correct classification of residents’ active transportation. Both perceived and audited walkability were important predictors of active transportation. For audited walkability, we recommend more exploration of decision tree approaches, given their predictive utility and ease of translation into walkability interventions.

## 1. Introduction

Encouraging more walking is an attractive public health goal, given that walking is the most popular form of physical activity (PA) across genders, age groups, and fitness levels [1,2] and it often achieves healthy, moderate-intensity levels of exercise [3]. Unfortunately, many communities have not been designed to be supportive of walking [4]. Consequently, researchers are investigating “walkability”—the designs and policies that might better support walking or other active modes of transportation, such as cycling [5]. In the current study we focus on the use of a street that was renovated into a “complete street”—one designed to encourage active use by providing light rail transit stops, wider sidewalks, pedestrian amenities, and bike lanes.

We examine how neighborhood walkability relates to complete street use by residents from the surrounding neighborhood, with both resident-perceived walkability scales and trained rater-audited walkability measures. The perceived walkability scales employed were informed by a past confirmatory factor analysis of a common walkability scale. To examine audited walkability, we adopt fairly novel methodological approaches. First, we employ rarely-used fine-grained walkability audits, which are specifically designed to assess walkability and identify modifiable features that could improve walkability. Second, we use machine learning algorithms when associating walkability audits to active transportation on the complete street.

### 1.1. Defining Perceived and Objectively-Assessed Walkability

Perceived walkability involves self-reported perceptions of environmental walkability features for areas such as neighborhoods. The Neighborhood Environment Walkability Scale—Abbreviated (NEWS—A), a widely used survey employed in this study, includes items that assess residents’ ease of walking to transit stops, good-quality sidewalks and bike paths, interesting neighborhood sights, traffic hazards, and crime perceptions [6,7].

Within objective measures, researchers often choose between pre-existing data organized in large-scale geographic information system (GIS) data bases, such as census tract street connectivity or residential density [8], and smaller scale audit data gathered by trained raters walking through the area and noting features such as crosswalks. We chose the Irvine–Minnesota Inventory (IMI) walkability audit, because it was developed with attention to past research and theory and is especially comprehensive, with 162 items representing four broad conceptual categories: pedestrian accessibility/infrastructure, pleasant aesthetics (which inventory creators called “pleasurability”), traffic hazards, and crime indicators [9,10].

Most studies suggest that perceived and audited walkability provide distinct measures of walkability, which should be treated separately. Perceived measures are not just a reflection of objective reality, given that people may not attend closely to environmental features, or past experiences and perceived social identities may inform their perceptions, or the social reputation of the area may affect perceptions [11], or people may interpret similar walkability features quite differently [12]. Audit scales also focus on many specific environmental features, which limit their correspondence with shorter, more general perceived walkability surveys. In addition, many audited items represent alternatives and one would not expect many of them to co-exist within one scale (e.g., crosswalks might be zebra-striped or yellow-lined, but not both). Indeed, weak relationships are often found between perceived and objective measures of particular environmental features: the presence [13] or proximity to neighborhood PA facilities [14,15,16], green space or parks in the neighborhood [17,18], hills [19], and distances to destinations [20,21]. Strong relationships are found when researchers deliberately sample low and high walkability neighborhoods [6,22] or create those comparisons by median splits of walkability [23,24]. However, the current study does not select neighborhood extremes and thus examines perceived and audited walkability separately.

### 1.2. Do Perceived and Objective Measures Relate to Physical Activity?

Research on whether walkability is associated with walking or PA is voluminous. Past reviews of perceived walkability correlates of walking suggest that perceived traffic safety, crime safety, land use mix, pleasantness of walking (e.g., lots of shade from trees on paths, sidewalks in good condition), and attractiveness [25,26] are the most consistent correlates. Another review found that perceived availability of physical activity facilities, sidewalks, shops, services, and traffic safety were all positively associated with PA [27]. For objective measures of walkability, one review found utilitarian walking (walking to destinations) was consistently associated with the presence and proximity of utilitarian destinations, such as local shops, services, and transit stops, and to sidewalks, while recreational walking was associated with recreational destinations and route aesthetics [28].

Studies that measure both objective and perceived walkability also show a variety of relationships to PA. These range from no relationships between reported PA and either perceived or audited objective walkability within a half-mile buffer (805 m) [29], to consistent relationships between PA and both perceived and GIS-based objective measures within a 2-km buffer [30] or a 1.6-km buffer [22] and to audited walkability within 400 m [31]. Sometimes, self-reported walking was related to perceived but not GIS-based objective walkability measures [32,33]. Another study found self-reported recreational PA was more strongly related to perceived walkability than to GIS-based objective walkability within an 800-m buffer, while self-reported commuting activity was more strongly related to the objective measures [34]. Other studies find objectively measured walkability correlates more strongly with PA than do perceived measures [35,36]. In sum, these studies suggest that both objective and perceived walkability can be important to PA outcomes, which makes it difficult to know how to boost walking unless both are measured.

Few studies have used the IMI audit, despite the fact that it was gauged to be the most comprehensive of the walkability audits [10]. One study using summary IMI scales found residents were more likely to use a new light rail stop if they lived on street blocks that had good walkability involving to crime safety, residential density, and land use diversity scales [37]. However, in multiple communities in Alberta, Canada, only 5 of 36 walkability scales related to self-reported walking, often in unexpected ways. In contrast, many individual IMI items were significantly related to walking. The researchers cautioned that we may not understand how best to create audited walkability scales and their results suggested that it is worth exploring relationships for individual IMI items [38]. Similarly, the creators of the IMI, in a study in Minneapolis and Saint Paul, Minnesota, used individual IMI items, finding that 16 of the 162 IMI items, such as the presence of sidewalks and pedestrian crossings, related to objectively measured PA or self-reported walking [39]. These studies suggest that researchers should explore ways to connect individual IMI items to active transportation.

The studies reviewed above also have several limitations. They typically do not include both perceived and objective indicators of walkability, despite much research demonstrating that both are useful. When both types of measures are used, they often have not been chosen to assess comparable concepts, such as relating personal safety to food and recreational destinations in the neighborhood [30]. Furthermore, self-reported PA is often used, despite research demonstrating that self-reported measures overstate PA compared to accelerometer measures [40]. When objective PA measures are used, it is often not clear whether the PA occurred in the place measured for walkability, such as when PA is assessed with total accelerometer PA measures but walkability is assessed only for the neighborhood [39]. When comprehensive audits of environmental walkability are used, they often rely on summary scales that can obscure the importance of individual items. When individual items are chosen, the statistical approach taken may not be designed to capitalize on rich and detailed measures. Finally, past research often does not address how far from home the active transportation is, despite evidence that people often limit walks to a length that ranges between approximately 0.80 km (half mile) to 1 km [41,42,43].

To overcome these limitations, we use global positioning system (GPS) and accelerometer evidence of active transportation along an improved commercial street central to the neighborhood. The data have already demonstrated that residents living closer to the complete street corridor are more likely to have active transportation trips there than more distant residents, consistent with the idea that the closest residents are most exposed to the street improvements [43]; thus we use an interaction of perceived walkability measures with distance from the corridor. We expect perceived walkability to have stronger relationships to active transportation on the complete street for residents living within the traditional walkable distance (up to about 1 km). After using the perceived walkability items to guide our selection of conceptually related environmental audit items from the IMI, we use a machine learning technique rarely used in walkability studies to develop a decision tree that identifies major walkability audit groups and then test those groups for differences by active transportation and distance to the complete street corridor.

In sum, we test three research questions:

1. Does perceived walkability relate to active transportation on the complete street corridor and does this relationship vary by distance to the corridor?
2. Does audited walkability relate to active transportation on the complete street corridor, and do walkability profiles vary by distance to the corridor?
3. Does perceived walkability relate to major walkability audit groups?

## 2. Materials and Methods

### 2.1. Data

Data for this cross-sectional study are drawn from phase two of the Moving Across Places Study (MAPS) in Salt Lake City, Utah, USA. MAPS is an evaluation of a complete street renovation involving approximately 4.2 km of improvements intended to attract active travelers, including five new light rail stops, a bike lane, and widened sidewalks. Phase one, in 2012, had examined pre-construction conditions [44], but the current study focuses on phase two, after construction of the new complete street corridor in April, 2013. From May to November of 2013, participants wore accelerometers (Actigraph GT3X+, Actigraph, Pensacola, FL, USA) and GPS loggers (GlobalSat DG-100 data loggers, GlobalSat, New Taipei City, Taiwan) for approximately 1 week. Participants completed surveys and were fitted for the devices at home and, approximately one week later, devices were collected.

### 2.2. Sample

During phase one, adult participants were recruited from randomly sampled blocks and were selected if they: lived within 2 km of the complete street, were over 18, could walk a few blocks, intended to stay in the neighborhood for more than 1 year, were not pregnant, were able to speak English or Spanish, and agreed to wear devices and fill out the surveys. We only recruited those who expected to stay in the neighborhood because we were following up the sample one year later in phase two; we included the 536 participants who participated in phase two for this study. Pregnant participants were excluded because they might have had difficulties with the waist-worn accelerometers and because their weight and possibly their active transportation would vary substantially over time due to pregnancy, not to their levels of active transportation. To be retained from phase one, participants needed to have accelerometer data from ≥ three 10-h days, and valid GPS data. Informed consent procedures were approved by the first author’s Institutional Review Board.

### 2.3. Measures

#### 2.3.1. Perceived Walkability

Residents’ perceived walkability of their neighborhood was assessed with the Neighborhood Walkability Scale—Abbreviated (NEWS—A), a 54-item survey [6]. Past research has used confirmatory factor analysis (CFA) to identify a 21-item subset, which we used as a starting point for our scale construction. The original CFA identified seven walkability scales: residential density, land use mix-diversity, street connectivity, walking/cycling facilities, aesthetics, traffic safety, and crime safety [7]. In order to achieve factors that provide good model fit, the current study supplemented the NEWS—A with 6 additional questions on: housing density (*n* = 1), land use mix-diversity (*n* = 1), crime safety (*n* = 3), and walking/cycling facilities (*n* = 1) for a total of 62 perceived walkability items. After confirmatory factor analyses, described in the Results section, we retained 20 items that were loaded on 6 factors.

#### 2.3.2. Audited Walkability

Neighborhood-wide walkability audits for all neighborhood streets were based on the Irvine Minnesota Inventory (IMI, *n* = 162 items), which uses as the unit of analysis a street segment—both sides of a street between intersections. For each participant, length-weighted street segment audits were averaged across the quarter-mile (approximately 0.40 km) street network buffer around each participant’s address, a common metric for representing the neighborhood [31,45,46]. The intraclass correlation, as a measure of interrater reliability, was 0.74, which is characterized as the top of the “good” range, specified as an intraclass correlation coefficient (ICC) of 0.60 to 0.74 [47].

The inventory authors suggested the IMI items could be organized into four conceptually distinct domains: accessibility, pleasantness (originally called pleasurability), perceived safety from traffic, and perceived safety from crime [9]. Good pedestrian accessibility provides access along sidewalks and supports for crossing roads, such as crosswalks. Pleasantness includes good views and comfortable facilities for pedestrians, such as street trees and front porches. Traffic hazards include features that create physical and/or psychological barriers to active transportation, such as high speed limits, absence of bike lanes, and many lanes of traffic. Crime indicators include features such as graffiti, litter, and poor street lighting.

To enhance comparability between perceived and audited walkability, 40 IMI items were chosen that were judged by three co-authors to be similar to the conceptual categories represented by the final 20 perceived walkability items. The IMI, like other walkability audits, does not provide items with one-to-one correspondence to the perceived walkability items, so the comparability is only approximate. For example, the NEWS—A scale of street connectivity is a concept that refers to a larger scale than a street segment, so it was not measured with the IMI, which focuses on conditions within each block. Similarly, the NEWS-A items regarding accessibility require an assessment of perceived distances from home to various destinations, which refers to relations across street segments so is not measured by within-segment assessments. Thus, the IMI variables emphasized accessibility or ease of walking for pedestrians within street segments, such as the presence of a sidewalk. Furthermore, particular physical features may represent multiple categories, which prevents simple item-to-item correspondence. For example, the original codebook for the IMI notes that bike lanes could be coded as both traffic and access features [48].

Consistent with some past research, IMI items were dichotomized to represent walkability using one of three coding schemes: 0 = neutral and 1 = good walkability; or −1 = poor and 1 = good walkability feature; or 0 = neutral and 1 = good walkability feature [39,49].

#### 2.3.3. Active Transportation on and Distance from the Complete Street

Mapped GPS and accelerometer data were used to create a dummy variable indicating that the participant engaged in active transportation on the complete street, based on speed and acceleration from GPS points merged with accelerometer counts. The company Geostats (now Westat) was contracted to integrate the GPS and accelerometry data so that we could identify the places for all trip stages that involved active transportation. A trip involving active transportation, defined as walking, biking, running, using bus, or using rail transit was considered to be on the complete street if the trip had any GPS points registering within a 40-m buffer from the street centerline. GIS measures of distance between home and the complete street were measured and expressed in 100 m increments. On average, participants lived 9.68 hundred m (in 100 m increments, thus 968 m) from the complete street.

#### 2.3.4. Control Variables and Sample Description

Given past research that relates some demographic variables to walkability [50] or physical activity [40], including walking [51,52], we controlled for gender, Hispanic ethnicity, and household income. We also controlled for having access to a car. If a participant had missing data on household income, it was imputed using regression imputation. Age was initially included as control variable for conceptual reasons; however, multicollinearity checks revealed that it was collinear with having access to a car. Car access was retained because it was significantly correlated with the outcome of active transportation on the complete street (Spearman *r* = −0.19, *p* < 0.001 for having a car, *r* = 0.05, *p* = 0.23 for age). Participants were on average 42 years old, with $42,000 U.S. dollars in household income. In addition, 51% were female, 25% were of Hispanic ethnicity, and 87% had access to a car and 31% (*n* = 167) used active transportation on the complete street.

### 2.4. Data Analysis Procedures

First, confirmatory factor analyses guided the development of perceived walkability factors, using IBM’s SPSS AMOS (analysis of a moment structures) version 22 [53]; resulting factors then guided the selection of IMI counterparts. To investigate how active transportation related to perceived walkability scales developed from a confirmatory factor analysis, and whether distance from the complete street moderates effects of walkability, logistic regressions (SPSS v22) were employed [54]. Predictors included perceived walkability scales, interactions between distance and perceived walkability, and control variables. Interaction tests involved standardized (z-scored) walkability scales interacted with centered distance from the complete street corridor. Tests revealed unacceptable levels of collinearity between perceived traffic hazards and crime indicators (condition indices > 5 with two individual coefficients greater than 0.5) [55]. To reduce collinearity without collapsing across scales, separate analyses of each of the five walkability scales were conducted with Bonferroni-corrected significance levels (0.05/5 = 0.01). The interactions between walkability scales and distance were tested with the Johnson–Neyman (J–N) technique, using the PROCESS macro [56] to identify and plot regions of significant interactions.

To investigate how active transportation related to individual audited walkability items, decision trees were created using R’s [57] rpart package [58]. The decision tree [59] classifies the subset of 40 IMI items and the control variables (gender, Hispanic ethnicity, household income, and car access) into meaningful splits along variable values, based on joint associations with active transportation on the complete street. Once meaningful splits were identified, terminal nodes (nodes that are at the end of decision tree splits) were used to identify groups of participants with similar underlying IMI features. Post hoc tests from one-way ANOVA demonstrated how these groups differed with respect to active transportation on the complete street and distance from the complete street. To summarize efficacy of the decision tree approach for this data set we calculated the percent of correct predictions of active transportation along the complete street (i.e., those predicted to use/not use active transportation vs. those who did/did not use active transportation).

## 3. Results

### 3.1. Perceived Walkability Scale Creation and Audited Walkability Item Selection

Perceived walkability scale creation started with an attempted replication of confirmatory factor analysis (CFA) scales of the NEWS—A items identified by Cerin et al. [60]. Cerin et al. created 6 factors from 21 items: access (*n* = 3), street connectivity (*n* = 2), infrastructure for walking/bicycling (*n* = 6), aesthetics (*n* = 4), traffic hazards (*n* = 3), and crime indicators (*n* = three single-item scales instead of one overall scale). A direct replication of this model yielded an unacceptable model fit: (χ² (364) = 2906.86; *p* < 0.001, comparative fit index (CFI) = 0.67, root mean square error of approximation (RMSEA) = 0.06, Tucker Lewis index (TLI) = 0.58, Aikake information criterion (AIC) = 3186.86) [61,62]. Due to poor model fit and the conceptual limitation of having three single-item crime factors, a modified version of Cerin’s CFA models was tested. Crime is believed to be important for walkability [44,63], thus we added three additional perceived crime items and created one overall crime factor. Acceptable model fit (χ² (339) = 845.37; *p* < 0.001, CFI = 0.91, RMSEA = 0.04, TLI = 0.90, AIC = 1087.37) was obtained for this study across 6 factors for the 20 items described in Table 1. Factors included access (*n* = 3), street connectivity (*n* = 3), infrastructure (*n* = 3), aesthetics (*n* = 3), traffic hazards (*n* = 3), and crime indicators (*n* = 5). Correlations among factors are displayed in Table S1 and correlations among individual items that load onto factors are displayed in Table S2. IMI items were selected by three authors, based on consensus judgment that the items represented one or more of the concepts measured by the NEWS—A concepts (see Table S3 for individual item descriptions).

### 3.2. Do Perceived Walkability and Distance from the Complete Street Relate to Active Transportation?

When testing interactions with distance recall that residents lived up to 2000 m from the complete street, with an average of 968 m. The logistic regressions used a centered version of the distance variable; however, in order to provide clearer interpretation of the results in the figures, we transformed distances back into meters from the complete street.

As shown in Table 2, perceived walkability did not have significant main effects but did show the expected walkability by distance interaction effects in three of five walkability scales (aesthetics, traffic and crime indicators; perceived infrastructure was significant at the *p* < 0.05 level but not significant at the required *p* < 0.01 Bonferroni-corrected alpha level). Also as expected, in all cases living closer to the complete street related to active transportation on the street.

**Figure 1:**
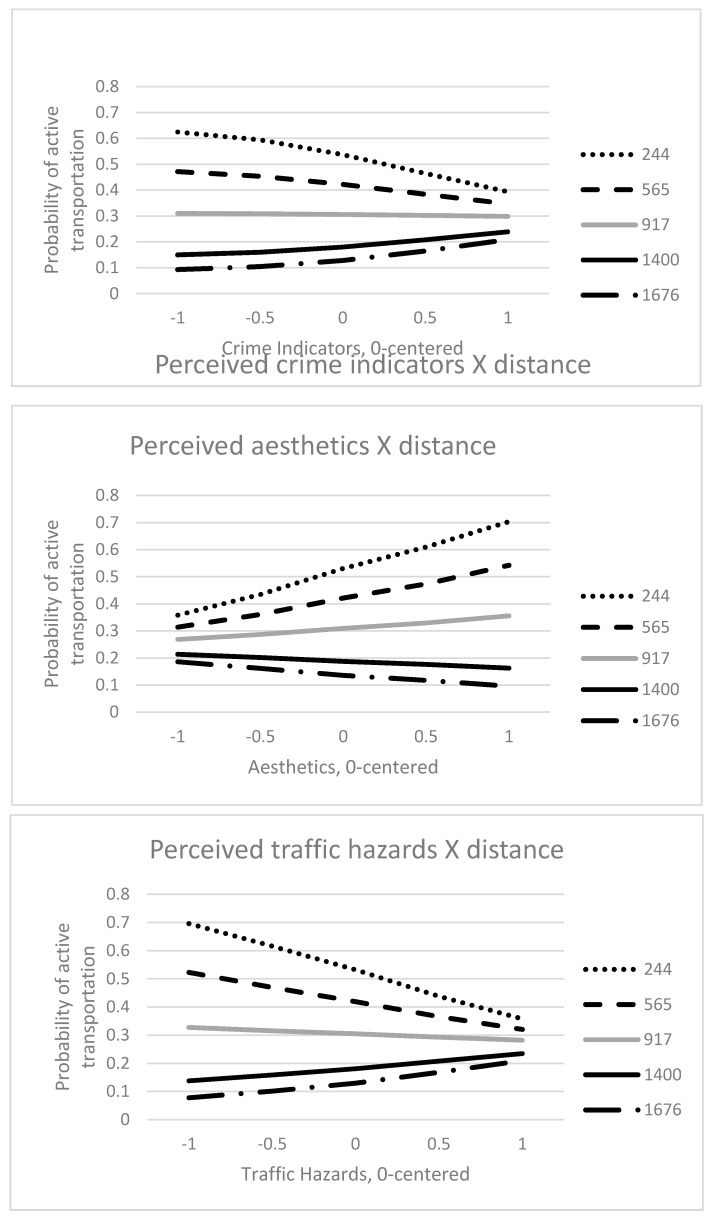
Plots of interactions between perceived walkability scales and distance from the complete street. *Note*. Legend represents distance from the complete street in meters.

For perceived aesthetics, among residents living closer to the complete street (ranging from 27 to 806 m, *p* < 0.05), the greater the pleasant aesthetics the greater the likelihood of active transportation on the complete street. Interaction effects are illustrated in Figure 1.

For perceived traffic hazards, residents living close to (27 to 721 m from the complete street, *p* < 0.05) and far from (>1543 m, *p* < 0.05) the complete street had different patterns. For residents closest to the complete street, more perceived traffic hazards related to lower likelihood of active transportation on the complete street. For more distant residents more perceived traffic hazards related to increased likelihood of active transportation on the complete street. Similar results were found for crime indicators. For residents close to the complete street (27 to 429 m, *p* < 0.05), greater perceived crime indicators associated with lower likelihood of active transportation on the complete street. For residents far from the complete street (>1840 m, *p* < 0.05), greater crime indicators associated with higher likelihood of active transportation on the complete street.

### 3.3. Does Audited Walkability in a Decision Tree Analysis Relate to Active Transportation?

**Figure 2:**
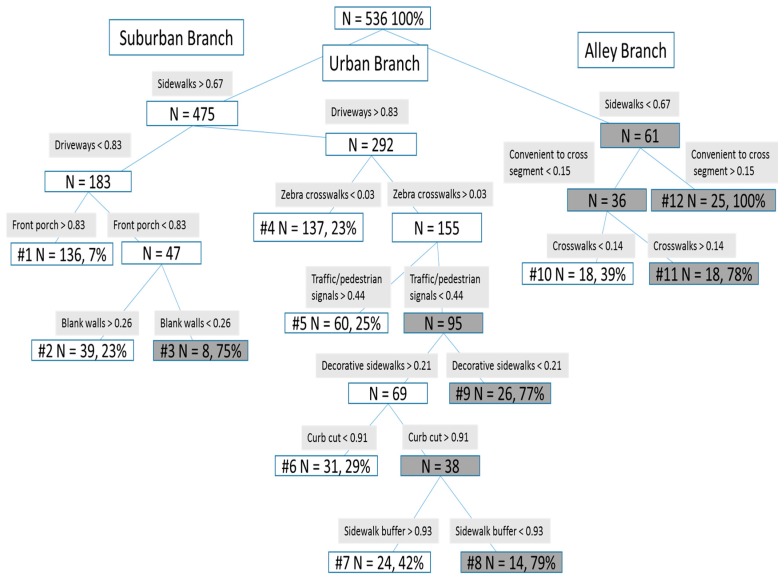
Decision tree results. Note. Grey nodes indicate predicted active transportation on the complete street. Percent in terminal nodes indicates percent of participants in that node who had global positioning system (GPS)-verified active transportation on the complete street.

A decision tree was calculated using as inputs the 40 individual audited IMI items (from Table S3) along with control variables, with the outcome variable being active transportation on the complete street. Figure 2 illustrates the decision tree results graphically and Table 3 summarizes the results textually. Three conceptual branches emerged that we described as a suburban, urban, and alley branches. These names reflect the underlying structure of the sample area, although there is substantial variability within some of the areas (see the description of 6 urban clusters below).

The suburban branch consists of features that often describe suburban areas. The largest group (*n* = 136) had street segments with many front porches, complete sidewalks, but relatively few (<0.83) driveways (compared to some urban nodes, as described below). There were two smaller groups of suburban residences that were similar to the largest group, except that relatively high (>0.26) or low (<0.26) levels of many blank walls were present. Although one might think of blank walls as belonging to big box stores or warehouses, windowless garage walls and walls around apartment complexes in this neighborhood also constitute blank walls.

The urban branch consists of 6 different clusters of street segments, showing greater diversity of street conditions that associate with active transportation to and along the complete street. Features that characterize urban areas often were defining features of these clusters. For example, defining features include relatively high levels of traffic/pedestrian signals (node 5), zebra-striped crosswalks (nodes 5–9), curb cuts (nodes 7 and 8), sidewalks buffered from the street traffic (node 7), and decorative sidewalks (node 8). Some of the urban clusters are quite similar and vary only along one feature. For example, street segments within nodes 7 and 8 share relatively high levels of having sidewalks, decorative sidewalks, driveways, zebra-striped crosswalks, and curb cuts and relatively low levels of having traffic/pedestrian signals. However, node 7 has an especially high levels of having sidewalks buffered from the street whereas node 8 has lower levels of having sidewalks buffered from the street. Some features are common across urban nodes, and serve to distinguish them from other branches. For example, the urban nodes all have very high levels of driveways attached to buildings, compared to the suburban nodes where driveways are still found on a majority of segments but at a lower level than for urban areas (i.e., <0.83).

The alley branch consists of areas where the roads are not “back alleys” but rather narrow and alley-like front roads that often belonged to distinctive areas such as a mobile home park or a compactly designed subdivision or apartment complex. Features distinctive to these areas include the only area with a low levels of sidewalks (node 12), which was a salient feature of some of the mobile home parks in this sample. The streets in this node were also different from those in all other nodes in that they were judged to have streets that are convenient to cross; this assessment is consistent with the lack of through traffic in such complexes and the narrowness of the streets in this area. The other alley nodes differed in that one group had more and one group had fewer crosswalks.

In the decision tree approach, 85 were correctly predicted as users and 347 were correctly predicted as non-users, leading to 432 correct predictions out of 536 or 80.6% correct (for incorrect predictions, 22 were predicted as users and 82 as non-users).

To clarify how the terminal nodes in the decision trees related to our key variables of active transportation on the complete street as well as to distance from the complete street, we conducted one-way ANOVA across the 12 terminal node groups, employing Tamhane’s post-hoc tests, useful for unequal sample sizes [64]. In Table 3 superscripts indicate significant differences between terminal nodes by active transportation on and distance to the complete street. The results show that there are multiple differences both across and within branches in terms of distance to the complete street and percentage engaging in active transportation on the complete street.

Further reflections on the patterns of results demonstrate several conclusions for this data set. First, only a handful of IMI features distinguish the groups. In fact, only 11 of the 40 IMI features helped to define and discriminate among groups. Second, many groups that had greater than average rates of active transportation were represented by a complicated range of intuitive and counter-intuitive walkability features. For example, all 25 residents in node number 12 used active transportation on the complete street and they had convenient-to-cross streets but relatively few sidewalks, which characterized a mobile home community close to the complete street. More intuitively, suburban node 1, had pleasant suburban features of porches, (relatively) few driveways, and many sidewalks and had residents who were the most distant from and least likely to use active transportation on the complete street. As in the earlier analysis of perceived walkability, closer distances related to higher likelihood of active transportation on the complete street even in the face of some less walkable features. Third, each of the suburban, urban and alley branches included nodes that differed in their residents’ tendencies to engage in active transportation on the complete street. Thus, the particular details of walkability items and distances to the complete street make a difference in predicting its use.

### 3.4. Does Perceived Walkability Relate to Major Walkability Audit Groups?

To understand walkability perceptions underlying the three groups defined by audited walkability, nonparametric correlations were calculated (see Table 4). The suburban residents perceived fewer crime indicators and traffic hazards and more street connectivity and pleasant aesthetics. In contrast, the urban residents perceived more crime indicators and traffic hazards and fewer aesthetics. The alley residents had no significant correlations.

**Figure 3:**
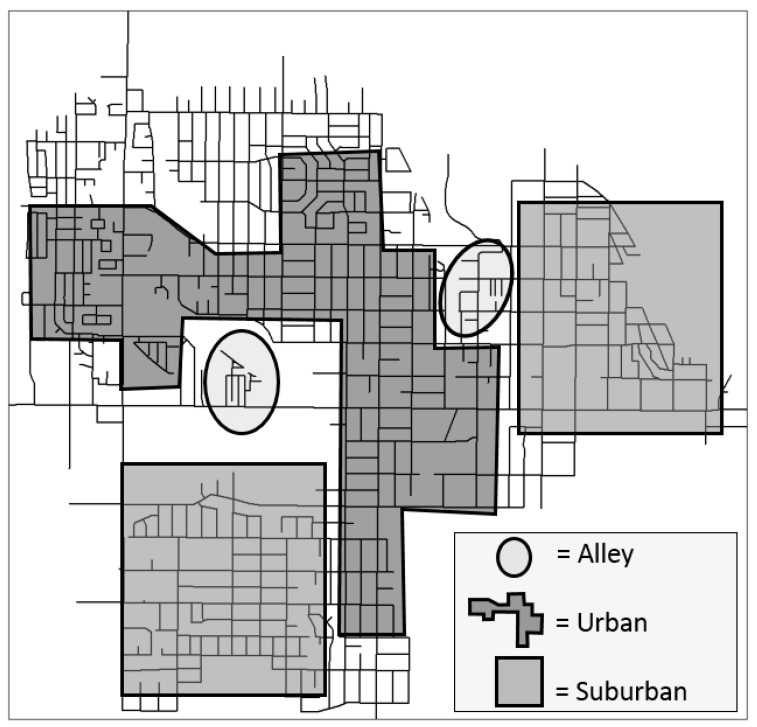
Study area map of branches from decision tree.

The three branch areas were mapped in Figure 3, using the sp package in R, with approximate locations shown. The urban corridor represented by the complete street (North Temple Street) is the east-west line that bisects the map, with many commercial and business services existing among blocks that straddle this street, along with residential dwellings. The other land area contributing to the urban branch is from blocks straddling the major north-south intersecting corridor, which is just west of the central business district of Salt Lake City. As noted in Table 3, the suburban areas are located the farthest from the main commercial corridors. This would be consistent with residents’ reports that these areas have more positive aesthetics and lower crime and traffic hazards. In contrast, the urban branch, generally close to the complete street and the major north/south intersecting corridor, reports lower aesthetics and more traffic and crime indicators. The smaller alley group, with compactly designed areas, incorporates most of the mobile home park residential areas and subdivisions of apartment complexes that have distinctive alley-like interior roads, which may not be lined by sidewalks.

## 4. Discussion

The current study represents one of few studies that can verify, with GPS data, whether participants used active transportation within a targeted geographical area. Perceived walkability interacted with residential distance from the complete street to achieve significant associations with active transportation, especially for those living near the street. Audited walkability showed that relatively few key features are needed to predict active transportation but that 12 patterns of street walkability features characterized the area, suggesting that walkability can be difficult to summarize neatly for a single area.

For nearby residents, results of the perceived walkability analyses are intuitive: better perceived aesthetics and infrastructure and fewer traffic and crime hazards relate to more active transportation on the complete street. There are counter-intuitive results are for those living farther than 1500 m from the complete street. At that distance, perception of more neighborhood crime and traffic hazards relates to more use of the complete street. Perhaps trips along the complete street represent an escape from or desire to avoid the poor perceived walkability qualities closer to home. Some researchers have suggested that enhancing perceptions of walkability is needed, especially when residents living in highly walkable neighborhood, but who perceived low walkability, were found to walk less than their neighbors who perceived high walkability [65,66]. The current study clarifies that this strategy may only be effective within a limited range from home, but that within this range better perceived walkability related to more active transportation. Furthermore, the strong main effect of proximity to the complete street for active transportation on the complete street reinforces past research that makes the same point. The closest residents benefit most from exposure to walkable opportunities such as complete street improvements [43] and/or good perceived walkability.

Generally, minor adaptations of Cerin et al.’s perceived walkability scales were needed for this current study [60]. These perceived walkability scales have been usefully employed in hundreds of studies, including recent work in 12 different countries [67]. The current results add to the assessment that perceived walkability concepts have significant applicability across settings. In addition, the coalescence of a single perceived crime indicators into one scale may prove useful to future investigations; we suggest future researchers may want to add additional crime indicator items, such as the ones used in the current study.

This study also used a decision tree technique, a relatively novel approach to understanding how audited walkability items may be related to active transportation. Using a decision tree technique also offers a different approach than the more traditional approach of creating multi-item scales [37,38]. Decision trees can capture non-linear and hierarchical relationships between walkability and active transportation on the complete street. These differences allowed us to explore and identify the different regions of the neighborhood that had unique walkability situations (such as the three suburban, urban, and alley branches). Results show that of 40 items, only 11 emerged as significant in terms of decision street branch splits. Several common walkability features mattered, but in ways that spoke to unique local conditions. For example, nearby mobile home and apartment complex communities had relatively few sidewalks but were close enough to support active transportation on the complete street. Similarly, zebra-striped crosswalks were present in the urban sections that had average or higher levels of active transportation, but were not present in the suburban or alley areas. Such special crosswalk treatments may be reserved for areas that draw many pedestrians and support their walking or may in fact attract pedestrians. In the suburban areas, the two nodes with the fewest front porches related to more walking, perhaps suggesting the more densely developed suburban areas encourage active transportation. Thus, the decision tree approach allows consideration of how walkability features may vary in importance, depending on contextual features, such as distance to a complete street or degree of urbanization.

The use of the decision tree is a relatively novel approach to understanding how audited walkability relates to active transportation and may pinpoint small combinations of individual items that might support precise design interventions for walkability. It is well suited to research problems that have many potential and conceptually appealing predictors that may be related to each other in a variety of ways. Although the focus in the present study was in the use of the complete street area, future research could apply the same approach to the study of walks elsewhere in the neighborhood, such as walks to parks or around one’s home. Given that the decision trees yielded good predictive scores, we recommend that future research consider the use of decision trees when examining relationships between complex and comprehensive audited walkability items and active transportation. It is not clear whether decision trees will yield idiosyncratic results or similar results across areas and types of active transportation (e.g., for leisure or transportation), but if a few variables relate to active transportation, transportation and urban planners could benefit by having clear targets for walkability supports. The current study suggests that small combinations of features relate to more active transportation, including traffic/pedestrian signals, sidewalks, and decorative sidewalks. These features represent fairly low investment improvements in walkability conditions, if their effect is confirmed in future research.

### Strengths and Limitations

This study is limited by the cross-sectional data and the limited environmental variability likely to exist within one contiguous neighborhood. Although objective measures verified active transportation and place of travel, the use of the complete street is a very specific behavior, which is more feasible for those close to the complete street. The GPS measures allowed us to verify the use of the complete street in relation to perceived and audited walkability around participants’ homes. Better geographic correspondence across measures could be attained in future research by, for example, by specifying a predefined area from home for perceived walkability measures and relating those measures to audited walkability and walking in those areas only. Furthermore, no self-reported motivations for each trip were gathered, limiting our ability to understand the purposes behind activities. We also used all the data present in the sample to identify patterns of relationships; future research could be strengthened by cross-validating results across subsamples. More research is needed for urban planners and transportation engineers to find better ways to support and encourage active transportation in urban settings, especially when relationships may include counter-intuitive associations, as seen in this and other studies [68,69,70]. Finally, greater variability in locales and measures is encouraged for future research.

## 5. Conclusions

The findings in this study clearly indicate that there are connections between the environment and active transportation. By including both perceived and audited walkability, the current study provided a greater understanding of how both types of walkability relate to active transportation. Similarly, close proximities to the street destination were shown to override poor audited walkability in some cases and relate to better perceived walkability associated with active transportation. This result shows that pedestrians can endure less walkable conditions. Future research is needed in order to know whether improving poor conditions would attract even more pedestrians. The more we understand the relationships between the environment and physical activity, the more we can promote healthy living with increased amounts of physical activity and active transportation.

## Figures and Tables

**Table 1:** Neighborhood Environment Walkability Scale—Abbreviated (NEWS—A), expanded: Descriptive statistics and factor loadings for six factors.

NEWS—A Items 1–17, with Additional Crime Items 18–20	Mean	Standard Deviation	Residual Errors	Factor Loading
Access				
1. Stores are within easy walking distance of my home	3.24	0.82	0.42	0.61
2. There are many places to go within easy walking distance of my home	3.11	0.91	0.23	0.85
* 3. It is easy to walk to a transit stop (bus, light rail) from home	3.60	0.71	0.43	0.36
Street connectivity				
4. The distance between intersections …is usually short…	2.82	0.93	0.70	0.45
5. There are many alternative routes for getting from place to place…	3.20	0.84	0.46	0.60
* 6. The streets… have few if any, cul-de-sacs (dead-end streets)	2.80	1.05	0.98	0.31
Infrastructure				
7. My neighborhood streets are well lit at night	2.69	0.92	0.34	0.77
8. Walkers and bikers on my neighborhood streets can be easily seen by people in their homes				
	2.99	0.82	0.36	0.67
* 9. There are crosswalks and pedestrian signals to help walkers cross busy streets…				
	3.09	0.89	0.59	0.51
Aesthetics				
10. There are many interesting things to look at while walking…	2.90	0.89	0.26	0.81
11. There are many attractive natural sights…	2.63	0.93	0.24	0.85
* 12. There are attractive buildings/homes…	2.66	0.90	0.32	0.78
Traffic hazards				
13. There is so much traffic along nearby streets that it makes it difficult or unpleasant to walk…				
	2.19	0.84	0.44	0.60
14. The speed of traffic on most nearby streets is usually slow (30 mph or less) (reversed)				
	2.17	0.93	0.74	0.38
* 15. Most drivers exceed the posted speed limits while driving…	2.83	0.87	0.64	0.38
Crime indicators				
16. There is a high crime rate…	2.41	0.89	0.42	0.68
17. The crime rate…makes it unsafe to go on walks at night	2.43	0.98	0.54	0.64
18. Gang activity	0.00	1.00	0.32	0.82
19. Groups of teenagers or adults hanging out … causing trouble	0.00	1.00	0.35	0.81
* 20. House or place you suspect drug dealing occurs	0.00	1.00	0.40	0.78

Note: NEWS-A = Neighborhood Environment Walkability Scale Abbreviated. *n* = 536. Response choices for items 1–17: (1) strongly disagree (2) somewhat disagree (3) somewhat agree (4) strongly agree. Z-scores were used for items 18–20. Instructions for responding to items 18–20: “Please rate the following problems you might have seen in this area in the last 12 months” ratings ranged 1 = no problem 10 = big problem. Factor loadings are standardized and were determined using AMOS Graphics version 22. * are used to denote which variable was the marker variable for each factor.

**Table 2:** Perceived walkability and distance associated with active transportation to the complete street in 2013: logistic regression models.

Predictor variables	Odds Ratio (R^2^)	95% Confidence Interval	*p*
Access	1.07	(0.87, 1.32)	0.50
Distance	0.88	(0.84, 0.91)	0.001
Access X Distance	0.99	(0.95, 1.03)	0.57
Nagelkerke R^2^	(0.18)		
Infrastructure	1.18	(0.96, 1.46)	0.12
Distance	0.88	(0.84, 0.92)	0.001
Infrastructure X Distance	0.95	(0.91, 1.00)	0.03
Nagelkerke R^2^	(0.20)		
Aesthetics	1.13	(0.91, 1.39)	0.27
Distance	0.87	(0.84, 0.91)	0.001
Aesthetics X Distance	0.95	(0.91, 0.99)	0.01
Nagelkerke R^2^	(0.20)		
Traffic hazards	0.96	(0.78, 1.17)	0.67
Distance	0.87	(0.83, 0.91)	0.001
Traffic hazards X Distance	1.07	(1.03, 1.11)	0.001
Nagelkerke R^2^	(0.20)		
Crime indicators	1.01	(0.82 , 1.23)	0.96
Distance	0.87	(0.84, 0.91)	0.001
Crime indicators X Distance	1.05	(1.01, 1.09)	0.01
Nagelkerke R^2^	(0.19)		

Note: Bonferroni-corrected significance levels are used (0.05/5 = 0.01). All analyses controlled for gender, Hispanic ethnicity, car access, and household income.

**Table 3:** Decision tree results.

Branch and Terminal Node Number and Description		N	AT%	Mean Distance
Node	Suburban branch			
1.	Front porch > 0.83, driveways < 0.83, sidewalks > 0.67	136	7 ^4,8,9,11,12^	1366.45 ^2,4–12^
2.	Blank walls > 0.26, front porch < 0.83, driveways < 0.83, sidewalks > 0.67	39	23 ^8,9,11,12^	688.55 ^1,4,5,11,12^
3.	Blank walls < 0.26, front porch < 0.83, driveways < 0.83, sidewalks > 0.67	8	75	1245.7412
	Urban branch			
4.	Zebra crosswalks < 0.03, driveways > 0.83, sidewalks > 0.67	137	23 ^1,8,9,11,12^	1123.83 ^1,2,6–12^
5.	Traffic/pedestrian signals > 0.44, zebra crosswalks > 0.03, driveways > 0.83, sidewalks > 0.67	60	25 ^8,9,11,12^	1105.56 ^1,2,6–12^
6.	Curb cut < 0.91, decorative sidewalks > 0.21, zebra crosswalks > 0.03, driveways > 0.83, sidewalks > 0.67	31	29 ^9,11,12^	464.75 ^1,4,5,7,8,10,12^
7.	Sidewalk buffer > 0.93, curb cut > 0.91, decorative sidewalks > 0.21, traffic/pedestrian signals < 0.44, zebra crosswalks > 0.03, driveways > 0.83, sidewalks > 0.67	24	42 ^1,2^	739.21 ^1,4–6,9,11,12^
8.	Sidewalk buffer < 0.93, curb cut > 0.91, decorative sidewalks > 0.21, traffic/pedestrian signals < 0.44, zebra crosswalks > 0.03, driveways > 0.83, sidewalks > 0.67	14	79 ^1,2,4,5^	739.6 ^1,4–6,9,11,12^
9.	Decorative sidewalks < 0.21, traffic/pedestrian signals < 0.44, zebra crosswalks > 0.03, driveways > 0.83, sidewalks > 0.67	26	77 ^1,2,4–6^	401.25 ^1,4,5,7,8,10,12^
	Alley branch			
10.	Crosswalks < 0.14, convenient to cross segment < 0.15, sidewalks > 0.67	18	39 ^1,2^	774.1 ^1,4–6,9,11,12^
11.	Crosswalks > 0.14, convenient to cross segment < 0.15, sidewalks > 0.67	18	78 ^1,2,4–6^	297.85 ^1,2,4,5,7,8,10^
12.	Convenient to cross segment > 0.15, sidewalks < 0.67	25	100 ^4,8,9,11,12^	137.18 ^1–10^

Note: Superscripts in the active transportation% and mean distance columns indicate significant differences between that node and the other node numbers on active transportation and distance, respectively. AT = active transportation on the complete street.

**Table 4:** Nonparametric correlations between perceived walkability scales (NEWS—A) scales and three branches based on audited walkability (IMI).

NEWS—A Scale	Suburban Branch	Urban Branch	Alley Branch
Access	0.08	−0.02	−0.08
Street connectivity	0.14 **	−0.08	−0.08
Infrastructure	0.06	−0.09 *	−0.06
Aesthetics	0.25 **	−0.23 **	−0.01
Traffic hazards	−0.15 **	0.17 **	−0.04
Crime indicators	−0.17 **	0.21 **	−0.07

Note. * *p* < 0.05; ** *p* < 0.01. NEWS—A: Neighborhood Environment Walkability Scale—Abbreviated; IMI: Irvine Minnesota Inventory.

## Supplementary Materials

The following are available online at www.mdpi.com/10.3390/1660-4601/14/9/1014/s1, Table S1: CFA factor correlations; Table S2: Correlation matrix of variables used in confirmatory factor analysis; Table S3: Audited walkability items: coded so that high walkability = 1.
