# Supplementary material for: Active Transportation on a Complete Street: Perceived and Audited Walkability Correlates
Source: Int J Environ Res Public Health. 2017 Sep 5;14(9):1014. doi: 10.3390/ijerph14091014 (PMC5615551; doi:10.3390/ijerph14091014)
Supplement: Supplementary file 1 [file ijerph-14-01014-s001.pdf]

Table S1
CFA factor correlations

	Correlation:					
	Access	Street Connectivity	Infrastructure	Aesthetics	Traffic Hazards	Crime Indicators
Access	-					
Street Connectivity	0.49	-				
Infrastructure	0.36	0.59	-			
Aesthetics	0.44	0.61	0.62	-		
Traffic Hazards	-0.40	-0.42	-0.50	-0.55	-	
Crime Indicators	-0.10	-0.07	-0.39	-0.35	0.72	-

Note. Correlations are standardized correlations. All correlations > 0.10 are significant at the $p<.05$ level.

Table S2
Correlation matrix of variables used in confirmatory factor analysis

	Variable Number																			
	1	2	3	4	5	6	7	8	9	10	11	12	13	14	15	16	17	18	19	20
Access																				
1. Stores close	-	54	19	13	26	5	11	13	12	13	8	15	-8	-11	2	-6	-8	-6	-5	-7
2. Many places to go	54	-	30	8	23	8	19	15	18	32	30	28	-17	-17	-10	-17	-20	-12	-12	-10
3. Transit easy walk	19	3	-	6	11	1	12	2	16	11	6	11	-13	-9	6	-8	-6	-8	-4	-5
Street connectivity																				
4. Close intersections	13	8	6	-	24	19	6	12	12	14	18	13	-5	-14	6	1	1	-3	0	3
5. Alternative routes	26	23	11	24	-	9	19	25	25	26	20	16	-6	-8	1	-7	-5	-1	-5	-2
6. Few cul-de-sacs	5	8	1	19	9	-	7	8	16	12	7	14	-4	-3	4	-4	-5	-1	8	8
Infrastructure																				
7. Well lit at night	11	19	12	6	19	7	-	44	31	37	32	28	-12	-16	-11	-22	-21	-17	-16	-19
8. Pedestrians visible	13	15	2	12	25	8	44	-	29	25	24	20	-12	-10	0	-20	-18	-15	-11	-12
9. Crossing signals	12	18	16	12	25	16	31	29	-	25	25	21	-6	-9	-4	-9	-2	-10	-5	-8
Aesthetics																				
10. Interesting sights	13	32	11	14	26	12	37	25	25	-	68	53	-16	-23	-14	-24	-17	-20	-11	-16
11. Attractive sights	8	30	6	18	20	7	32	24	25	68	-	64	-12	-16	-13	-29	-19	-25	-18	-21
12. Attractive buildings	15	28	11	13	16	14	28	20	21	53	64	-	-23	-20	-16	-32	-31	-31	-21	-28
Traffic Hazards																				
13. Lots of traffic	-8	-17	-13	-5	-6	-4	-12	-12	-6	-16	-12	-23	-	23	25	21	27	24	21	20
14. Traffic speed slow	-11	-17	-9	-14	-8	-3	-16	-10	-9	-23	-16	-20	23	-	12	13	16	17	13	14
15. Cars speed high	2	-1	6	6	1	4	-11	0	-4	-14	-13	-16	25	12	-	19	20	16	11	12
Crime indicators																				
16. High crime rate	-6	-17	-8	1	-7	-4	-22	-20	-9	-24	-29	-32	21	13	19	-	63	48	42	49
17. Crime unsafe night	-8	-2	-6	1	-5	-5	-21	-18	-2	-17	-19	-31	27	16	20	63	-	40	38	40
18. Gang activity	-6	-12	-8	-3	-1	-1	-17	-15	-10	-20	-25	-31	24	17	16	48	40	-	66	60
19. Trouble groups	-5	-12	-4	0	-5	8	-16	-11	-5	-11	-18	-21	21	13	11	42	38	66	-	68
20. Drug dealing place	-7	-1	-5	3	-2	8	-19	-12	-8	-16	-21	-28	20	14	12	49	40	60	68	-

Note. Boldface indicates significance at p<.05 level. Leading zeros and decimals were removed to save space. Full variable wording in Table 1. Single digit numbers have a leading zero.

Table S3
Audited walkability items: coded so that high walkability = 1

1. Presence of sidewalk buffer
2. Presence of sidewalk
3. Presence of curb cut
4. Are sidewalks shaded by trees?
5. Are crosswalks present?
6. Presence of white painted lines at crosswalk
7. Presence of yellow pedestrian crossing sign
8. Presence of zebra striping at crosswalk
9. Presence of a pedestrian walk signal
10. Presence of a median
11. At least 50% of block has buildings
12. Presence of different road surface or paving
13. Presence of a traffic signal
14. Presence of a front porch
15. Presence of a detached single family home
16. Presence of flowers
17. Presence of buildings with garages
18. Absence of predominant driveways
19. Prominence of garages
20. Presence of street trees
21. Presence of historic buildings
22. Presence of decorative/unique sidewalk
23. Presence of townhome/condo with 3 units+
24. Speed limit above 25 mph
25. Presence of three or more lanes of traffic
26. Absence of a speed bump
27. Presence of a parking lot
28. Block is inconvenient to cross
29. Block is unsafe to cross
30. Low amount of parked vehicles along street
31. No bike lanes present
32. Absence of street and traffic signs
33. Absence of traffic/pedestrian signals
34. Blank walls are present on block
35. Closed view
36. Presence of litter
37. Presence of bars on windows
38. Presence of graffiti
39. Absence of outdoor lighting
40. Presence of freeway/overpass
